# COVID-19 Lockdown and Creativity: Exploring the Role of Emotions and Motivation on Creative Activities From the Chinese and German Perspectives

**DOI:** 10.3389/fpsyg.2021.617967

**Published:** 2021-10-28

**Authors:** Sebastian Hofreiter, Xiang Zhou, Min Tang, Christian H. Werner, James C. Kaufman

**Affiliations:** ^1^Institute for Creativity and Innovation, University of Applied Management, Ismaning, Germany; ^2^Department of Social Psychology, Nankai University, Tianjin, China; ^3^HSSH University Institute Schaffhausen, Schaffhausen, Switzerland; ^4^Triagon Academy, Marsa, Malta; ^5^Seeburg Castle University, Seekirchen am Wallersee, Austria; ^6^Neag School of Education, University of Connecticut, Storrs, CT, United States

**Keywords:** emotion and creativity, motivation and creativity, everyday creativity, COVID-19, lockdown, cross-cultural study, China, Germany

## Abstract

For much of 2020, countries around the world fought against the COVID-19 pandemic. Many countries went into lockdown to control the fast spread of the virus. The unusual restrictions and confinement of the lockdown brought about new challenges for people’s everyday lives. With flexibility, adaptability, and problem-solving at the core of its nature, creativity has the potential to help people cope with harsh and uncertain circumstances. Were people more, the same, or less creative in their everyday life during the period of lockdown, and in which ways? What are the emotions and motivations underlying their creative or non-creative behaviors? The current study aims to explore these questions from a cross-cultural perspective. A total of 754 comparable employee samples from three Chinese and three German cities were asked about their moods during the lockdown period, their self-rated level of creativity in daily lives before and during the lockdown, and their motivations behind their creative activities. Significant increases in creativity were observed in all everyday activities in both countries with only two exceptions in the German sample. Despite minor differences, a common pattern was found across cultures: whereas the activating positive mood could directly lead to the increase in creativity in some everyday activities, such a direct Mood-Creativity link was limited in the activating negative mood circumstances. In such circumstances, motivation intervened to enable the link to creativity. It was also found that this indirect effect of motivation between mood and creativity was more pronounced with the German participants.

## Introduction

The year 2020 will be remembered as a year full of unprecedented experiences due to the highly infectious and hitherto unknown COVID-19 virus. In order to control the fast spread of the pandemic, many countries around the world went into lockdown, closing public and entertainment places, limiting social contact, and restricting traveling and outdoor activities. Though these prevention measures have been proven to be effective in containing the fast transmission of the disease (e.g., Tian et al., 2020), the side effects, particularly the detrimental psychological effects such as increased depressive symptoms, anxiety, and severe stress of lockdown, are also obvious (for a review, see Brooks et al., 2020). In spite of this, the restrictions and uncertainty that lockdowns have brought about can also push people to unleash their creative potential to make sense of life (e.g., Glăveanu, 2010) and cope with uncertainty (Ford, 1996; Beghetto, 2019). The social distancing or isolation can give people time to “engage in long-forgotten hobbies, neglected passions and unfulfilled dreams” (Banerjee and Rai, 2020, p. 526).

As all cultural centers, entertainment places, sport arenas and, for a long time, schools were shut down, home became the *only* place for multiple purposes, be they social activities, entertainment, sports, or home-schooling. These unusual changes posed challenges to people‘s daily routines, requiring adaptation, compromise, or improvisations. Therefore, the current study investigates if participants’ everyday creativity increased or decreased during the lockdown across various domains. In addition, this study also aims to identify the underlying emotional and motivational psychological determinants of a possible change in everyday creativity caused by the COVID-19 lockdown. The present study focuses on the following questions: Were people more, the same, or less creative in their everyday life during lockdown, and in which ways? What were the emotions and motivations underlying their creative or non-creative behaviors? Since the pandemic and lockdown measure are a global phenomenon, we took a cross-cultural perspective investigating this research questions with data from China and Germany. The COVID-19 lockdown resulted in a largely unprecedented worldwide situation, resulting in few comparable scenarios to be used to base possible predictions. Most past studies on creativity’s relationship with emotions and motivations were conducted during traditional times, not moments of extreme crisis. Further, given the cross-cultural design of the study, we could not simply rely on existing theoretical frameworks based on Western, Educated, Industrialized, Rich, and Democratic (WEIRD) samples (Henrich et al., 2010). Therefore, we consider this study to be exploratory, designed to enable future replication and research, as opposed to relying on past work that would not be particularly comparable. As a result, to reflect such exploration, no direct hypotheses will be formulated.

### Confinement/Restriction and Everyday Creativity

Creativity is complex. There is a broad range of factors that influence people’s creativity, from interindividual components (e.g., cognitive strengths, personality, or motivation) to external components such as the social environment (Amabile and Pillemer, 2012). Following this point of view, a vast amount of research has shown beneficial or inhibiting contextual factors in the workplace (e.g., Amabile et al., 2004; Shalley et al., 2004), schools (e.g., Cole et al., 1999; Yi et al., 2013), and cultures (e.g., Maddux and Galinsky, 2009). Rehn and De Cock (2009) noted that the novelty aspect of the creative process can be based on the reduction or simplification of things. Thus, constraints don’t need to be harmful for creativity; indeed, they are incorporated in the concept of creativity itself (Sternberg and Kaufman, 2010). Across history, great creative achievements have occurred under constraint (Stokes, 2005). Haught-Tromp (2017) showed that the external or internal implementation of constraints can increase creativity across diverse creative tasks. Being confined, a more extreme form of restriction, is also shown to have influence on people’s creative behavior; however, malevolent forms of creativity can emerge from confinement (Singer, 2010). In times of COVID-19, many countries went into lockdown to control the fast spread of the virus. In both China and Germany, lockdown measures included the closure of cinemas, theaters, gyms, and other non-essential gathering places; implementation of traffic control; remote learning and working; and the encouragement of residents to reduce their outdoor activities and not participate in social gatherings. The unusual restrictions and confinement of the lockdown brought about various challenges to people’s daily lives. These included having less physical space for individual activities, fewer direct communications, daily travel restrictions, and new modes of work; all of these strongly influenced people’s experience, behavior, and attitudes (Sibley et al., 2020). With flexibility, adaptability, and problem-solving at the core of its nature, creativity has the potential to help people cope with harsh and uncertain circumstances.

This study focuses particularly on the influence of confinement and restriction on everyday creativity across a multitude of domains. Contemporary theories of creativity maintain that creativity exists in different domains (Kaufman and Baer, 2005) and at different levels, ranging from the Big-C (creative genius) and Pro-C (expert-level creativity) to little-c (everyday creativity) and mini-c (personal creativity) (Kaufman and Beghetto, 2009). It is worth noting that in the current study, everyday creativity is understood in a broad sense which includes both the “little-c” and “mini-c.” Whereas “little-c” creativity focuses on everyday creative activities that the non-experts laypersons would participate each day (such as cooking and drawing) (Richards et al., 1988), “mini-c” creativity refers to the novel and personally meaningful interpretation of experiences, actions, and events (Beghetto and Kaufman, 2007). The category of “mini-c” was included because given the novelty of the COVID-19 pandemic, people needed to interpret the unprecedented experiences, actions, and events happening to them.

There is no solid agreement on the best structure of creative domains. Studies investigating self-reported creative behavior and abilities often end up with different numbers and categories of domains (Ivcevic and Mayer, 2009; Kaufman, 2012; Diedrich et al., 2018; Benedek et al., 2019), most of which tend to include aspects of everyday, intellectual, STEM-related, and artistic (including visual, writing, and performance) creativity. It is worth noting that most of the above studies (and comparable scholarship) have been conducted in Western countries. As Kandemir and Kaufman (2019) suggest, creativity domain structures may vary in different countries. For example, Werner et al. (2014) administered the Revised Creativity Domain Questionnaire (CDQ-R) (Kaufman et al., 2009) to a Chinese sample and found that a five-factor model was a better fit than the existing four-factor model (established with an American sample). Given the novelty of the COVID-19 situation and the cross-cultural design, no existing creativity domain inventories would suit the current study. Therefore, we used a scale specifically developed for the purpose of the study through discussions with Chinese, German, and other colleagues. Details of the scale will be introduced in the “Materials and Methods” section.

### Affect and Creativity

Among the many predictors of creativity, affect is considered one of the most significant (Mumford, 2003; Baas et al., 2008). According to Frijda (1993), affect is the most general term for people’s subjective feelings which are embodied by long-lasting mood states or stimuli-triggered emotions. In the current paper, moods and emotions are used interchangeably. There is solid agreement that creativity benefits from a positive mood (e.g., Ashby et al., 1999; Davis, 2009). The effect of a negative mood on creativity is, however, plagued with inconsistent results showing the co-existence of the facilitating, inhibiting or non-effect of negative moods on creativity (for a review, see Baas et al., 2008). De Dreu et al. (2008) developed a dual pathway model to account for the mood-creativity link, conceptualizing affect through two underlying dimensions: valence (positive vs. negative) and activation (activating vs. deactivating). This model posits that activating moods (e.g., happy, elated, fearful, angry) lead to higher fluency and originality through increased cognitive flexibility. Deactivating moods (e.g., relaxed, serene, sad, depressed) lead to higher creativity through enhanced perseverance (Nijstad et al., 2010). The dual pathway model has been supported with empirical research (e.g., To et al., 2012; Yang and Hung, 2014; Montani et al., 2016; Karwowski et al., 2017). In terms of cross-cultural mood comparisons, people from Eastern cultures tend to express an emotion related to the group rather than themselves. Further, they exhibit mood dialectically, which is the opposite of Western cultures, where people exhibit free expression and private emotion (Spencer-Rodgers et al., 2010; Liu et al., 2016). Considering that both positive and negative affect might be increased during the COVID-19 pandemic (Lades et al., 2020), we predict that both activated positive and negative moods will contribute to creative activities during the COVID-19 lockdown period.

### Motivation and Creativity

If and how constraints influence creativity seems to be dependent on the motivational state and cognitive resources used by the different types of constraints (Roskes, 2015). There is a long tradition in the field of creativity to examine the influence of intrinsic vs. extrinsic motivation on creativity (e.g., Amabile, 1996; Collins and Amabile, 1999). Whereas intrinsic motivation is being engaged in activities for their very own sake (e.g., personal interest, task enjoyment), extrinsic motivation is driven by the prospect of potential rewards. A meta-analysis of 183 studies involving over 200,000 participants revealed that intrinsic motivation is a medium to strong predictor of performance (*p* = 0.21–0.45), including creativity (Cerasoli et al., 2014). However, extrinsic motivation such as incentives for performance can, under certain circumstances, also positively influence creative outcomes (Gerhart and Fang, 2015). Therefore, the dichotomy of intrinsic vs. extrinsic motivation needs to be examined to understand the nuanced influence of motivation on creativity. Several theories have explored this direction, including the hierarchical model of intrinsic and extrinsic motivation, which also incorporates the social and contextual factors (Vallerand, 1997); the approach vs. avoidance motivation distinction (e.g., Roskes et al., 2012); and proposed creative needs such as beauty, power, discovery, communication, individuality, and pleasure (Luria and Kaufman, 2017). The Two-Dimensional Framework of Motivation (Forgeard and Mecklenburg, 2013) broadens the traditional unidimensional view of motivation for creativity (i.e., intrinsic vs. extrinsic) to also include the intended beneficiaries (i.e., the self vs. others), enabling us to also look into the social aspects of the creative motivation.

This model was applied to guide the measurement of motivations for creativity during the COVID-19 lockdown in the current study. Since COVID-19 and the lockdown have had strong impacts on our relationships (Luchetti et al., 2020), different types of social-oriented (e.g., helping others) and beneficiary motivations (e.g., distraction or coping with negative emotions) might play an important role in engaging in creative activities during COVID-19.

### Mood-Motivation-Creativity Relationship

Both emotional and motivational processes influence human action (Bradley and Lang, 2007). However, emotions and motivations can be differentiated by their main focus (valence reaction vs. goal orientation and implementation) (Brewer and Hewstone, 2004). Despite their different core functions, both contribute to subjective feelings, thus influencing behavior (Batson et al., 1992; Berridge, 2018). Research suggests that affective states (i.e., mood) influence individuals’ motivation for action (Schwarz and Bohner, 1996). Gendolla (2017) describes emotions as a “hypo-phenomena,” influencing action through being a precondition of motivation. In the field of creativity, the Dual Pathway to Creativity Model (De Dreu et al., 2008, 2012) similarly postulates that the activation and arousal caused by different mood states influence motivation and thus the creative performance of individuals. Although the Mood-Creativity relationship is mainly linked to positive moods, the vast complexity and multi-dimensionality of creativity makes it necessary to expand the existing Mood-Creativity relationship (Kaufmann, 2003a); incorporating motivation is a good starting point. For example, negative mood states can increase people’s tendency to act or adapt to specific situations (Forgas, 2013). To et al. (2010) introduced the creativity-as-mood-regulation perspective emphasizing the role of goal orientation in the Mood-Creativity link of employees. The authors suggest that the influence of both negative and positive moods on creativity are stronger when a learning-goal orientation is active. Hence, it seems important to not only incorporate motivational orientations when evaluating the positive and negative mood states on creativity but also the intended purpose of the creative activity. Based on this literature and given the complexity and novelty of the COVID-19 situation, requiring people to learn and adopt to new expectations, we postulate both negative and positive moods to trigger different motivations for creativity which, in turn, will lead to different levels of creativity in different domains.

## Materials and Methods

### Participants

A total of 754 employees from China (*n* = 415, 51.8% females, *M*_*age*_ = 34.7, *SD* = 8.67) and Germany (*n* = 339, 52.5% females, *M*_*age*_ = 41.1, *SD* = 10.43) participated in the study. The Chinese and German participants were from different types of organizations, with over half of them from the branches of commercial services, health or social services, business organizations or production/manufacturing. The majority (94.9%) of the Chinese participants belonged to the Han ethnic group, who represent the overall ethnic proportion of the Chinese population. Table 1 presents a summary of the demographic information of the sample of the study. From this table we can see that the two samples are fairly comparable in terms of gender and occupation. The German sample is a bit older than the Chinese sample. The innovation level (including culture and infrastructural) of the city might influence people’s creative behaviors as there is evidence that social environment and climate play a key role in facilitating or impeding personal creativity (West, 2002; Hunter et al., 2007). Further, the lockdown measures in different regions and cities need to be considered as these differences can cause variations in people’s emotions, motivation, and creativity level. In order to control for the variations that regional differences can cause to the results, we applied three extra criteria to match the cities of residence of the participants: population, the rank in the Global Innovation Index, and the COVID-19 lockdown time and measures.

**TABLE 1:** Sample demographic information.

	China (*n* = 415)	Germany (*n* = 339)
**Gender**		
Male	200 (48.2%)	159 (46.9%)
Female	215 (51.8%)	178 (52.5%)
Others	0 (0%)	2 (0.6%)
**Age**		
Minimum	19	18
Maximum	63	68
*Mean*	34.7	41.1
*SD*	8.67	10.43
**Occupation**		
Employees	415 (100%)	339 (100%)
Others	0 (0%)	0 (0%)
**Industry**		
Commercial services	77 (18.6%)	82 (24.2%)
Health, social affairs	70 (16.9%)	50 (14.7%)
Business organizations	55 (13.3%)	40 (11.8%)
Production, manufacturing	45 (10.8%)	26 (7.7%)
Natural Science/IT	38 (9.2%)	33 (9.7%)
Transportation, logistics	23 (5.5%)	37 (10.9%)
Media Arts, Culture	16 (3.9%)	25 (7.4%)
Gastronomy, hotels, tourism	15 (3.6%)	27 (8.0%)
**Cities**		
	Shenzhen: 160 (38.6%)	Berlin: 121 (35.7%)
	Beijing: 143 (34.5%)	Hamburg: 115 (33.9%)
	Shanghai: 112 (27.0%)	Munich: 103 (30.4%)

Table 2 presents the detailed information of this comparison. From this table we can see that the three Chinese and German cities selected for the study are among the top 10 most populous and most innovative cities of the country and the official lockdown measures to prevent the spread of the COVID-19 virus in the cities were fairly similar.

**TABLE 2:** Comparability of the participating cities.

Country	Cities and number of participants	Population in 2019 (million)	Global Innovation Index 2019	Official lockdown measures against the spread of COVID-19
China (*n* = 415)	Beijing: 143 (34.5%) Shanghai: 112 (27.0%) Shenzhen: 160 (38.6%)	#3. (21.5)^1^ #2. (24.3)^1^ #7. (13.4)^1^	#2. (4.95)^3^ #3. (2.24)^3^ #1. (6.08)^3^	January 25 – February 21st, 2020: First level emergency response; Six public prevention guidelines for general purposes, tourism, family, public places, public transportation, home observation^4^
Germany (*n* = 339)	Berlin: 121 (35.7%) Hamburg: 115 (33.9%) Munich: 103 (30.4%)	#1. (3.70)^2^ #2. (1.85)^2^ #3. (1.48)^2^	#5. (0.76)^3^ #8. (0.37)^3^ #2. (1.05)^3^	March 22nd – April 20th, 2020: Exist and travel restrictions, cancelation of all major public and private events reduction of social contacts, home office recommendation, close of kindergartens, schools, universities, cinemas, restaurants, bars, theaters, etc.^5^

*#Stands for the rank in the country. Numbers in the parentheses are the values or percentages of the respective criterion.*

*^1^Chinese National Bureau of Statistics: http://data.stats.gov.cn/easyquery.htm?cn=E0105.*

*^2^German Federal Office of Statistics: https://www.destatis.de/DE/Themen/Laender-Regionen/Regionales/Gemeindeverzeichnis/Administrativ/04-kreise.html.*

*^3^Global Innovation Index 2019: https://www.wipo.int/publications/en/details.jsp?id=4434.*

*^4^Information published on the website of the Chinese Central Government: http://www.gov.cn/zhengce/2020-06/07/content_5517737.htm.*

*^5^Information published on the website of the German Ministry of Health: https://www.bundesgesundheitsministerium.de/coronavirus/chronik-coronavirus.html.*

### Procedures

Measurement equivalence is one important premise of cross-cultural studies (Hult et al., 2008). Procedures were taken in the present study, from research design to data analysis, to ensure that the data from China and Germany were as equivalent as possible. First, all research instruments were adapted to the COVID-19 lockdown situation, which provided the common context for the cross-cultural study. Second, the questionnaire, originally in English, was translated into Chinese and German by applying the team-based collaborative and iterative translation method (Douglas and Craig, 2007). The most convenient method of back translation (Brislin, 1980) was not used because of its weakness in assuring the conceptual equivalence and cross-cultural validity of the different versions of the instruments (Douglas and Craig, 2007). Two Chinese-German bilingual translators translated the questionnaire by strictly following the steps of the collaborative and iterative translation method. In case of discrepancies, the third author of the article, who is trilingual and has a psychological background, joined the discussion with the two translators until the best translation was agreed upon. Third, multiple rounds of pretests were conducted to ensure conceptual equivalence, measurement accuracy, and smooth data collection. Fourth, to ensure the comparability of the samples, we set a clear sampling frame for the study and matched the samples from both countries in terms of age, gender, occupation, and city of residence (see Tables 1, 2).

This study was approved by the Institutional Review Board of the University of Applied Management, Germany and that of the Nankai University, China. The participants of both countries have provided their written informed consent to participate in this study. Data were collected between July and August of 2020 in China and Germany using online survey tools (Wenjuan Xing in China and UniPark in Germany). Overall, the participants took about 10 min to complete the questionnaire. To ensure data quality, we applied the following procedures to clean the data: (1) As the pretests showed that at least 3 min were needed to complete the survey, participants who spent less than 200 s were excluded from analyses; (2) To avoid careless and untrustworthy responses, one attention check question was imbedded in the middle of the questionnaire (“If you see this question, please select “Extremely”). If a participant failed this check, his/her data were excluded from analyses; (3) To reduce social desirability, which is prone in self-report measures, we imbedded two honest response questions suggested by Vésteinsdóttir et al. (2019). All cases that failed this check were excluded from analyses.

### Data Analysis

To ensure the measurement equivalence, we used exploratory factor analysis (EFA) and confirmatory factor analysis (CFA) to identify and validate the factorial structure of each measurement in China and Germany. The sample was randomly split into two. Sample 1 (*n* = 286) was used for EFA, and sample 2 (*n* = 468) was used for CFA. Results of these analyses can be found in the Appendix. In the first step, EFA was conducted based on Eigenvalues > 1 (Kaiser, 1960) using Varimax or Oblimin rotation and the loading cut-off value of ±0.40 as recommended by Field (2013). Following the recommendation of Muthén (1997), we used pooled data based on random samples drawn from both countries for the EFA and CFA as the analysis of the interclass correlation coefficients (ICC) revealed that both sample resemble each other (ICC value was < 0.133; range: –0.002; 0.133). As the second step, we conducted CFA to test the model fit using root mean square error of approximation (RMSEA), standardized root mean square residual (SRMR), comparative fit index (CFI), and Tucker-Lewis Index (TLI). CFI and TLI values > 0.90 indicate an acceptable fit (Hu and Bentler, 1999) fit. For the RMSEA and SRMR values we followed the recommendation of Schermelleh-Engel et al. (2003) using a cut-off of 0.08 for an acceptable fit. Finally, we examined the cross-cultural equivalence of each measurement by checking for the configural and metric invariance of the measures. Results of the EFA and CFA tests are presented in the Appendix. After conducting the cross-cultural equivalence tests, we used SPSS and PROCESS to test our hypotheses using a correlation analysis, paired-sample *t*-test, hierarchical regression analysis, and mediation analysis.

### Measures

**The Impact of COVID-19** was measured by two items, one measuring the overall influence of COVID-19 on the participants’ everyday lives and the other its influence on their work. A 11-point Likert scale was used for with 0 meaning “no influence at all” and 10 meaning “extreme influence.”

**Positive and negative activating moods** were measured with three positive activating moods (PAM; enthusiastic, interest, and inspired) and three negative activating moods (NAM; upset, angry, and anxious). These items were drawn from the scale used in To et al. (2012), which originally included four positive and four negative activating items. In the current study, we dropped ‘‘excited’’ and ‘‘ashamed’’ from the scale on the basis of the pretest with both Chinese and German participants, who reported they did not see the relevance of these two items^1^. Participants used a 5-point Likert scale ranging from 1 (not at all) to 5 (very much) to indicate how much they experienced each mood during the lockdown period. EFA with Varimax rotation and the subsequent CFA confirmed the two-factor model of the construct with satisfactory model fit (see Appendix). The internal consistency of the measures was good in China (Cronbach’s α of 0.82 for PAM; 0.83 for NAM) and satisfactory in Germany (0.73 for PAM; 0.75 for NAM).

**Motivation for creativity** was measured with a scale developed based on the Reciprocal Model of the Creative Process proposed by Forgeard and Mecklenburg (2013). This model adds a social dimension (self-oriented vs. other-oriented beneficiaries) to the traditional locus of motivation (intrinsic vs. extrinsic) and proposed four types of motivation: Intrinsic-Self (Growth), Intrinsic-Others (Guidance), Extrinsic-Self (Gain), and Extrinsic-Others (Giving). A 24-item scale, 6 items for each type of motivation, were developed. Considering the special request that the lockdown has posed on people, namely to spend time with oneself (Banerjee and Rai, 2020), we replaced the “Guidance” dimension with an additional intrinsic-self motivation related to dealing with boredom, killing time, and seeking distraction. Participants reported on a 5-point Likert scale ranging from 1 (not at all) to 5 (extremely) the extent these possible motivators led them to engage in new and creative activities during the lockdown period. EFA with Oblimin rotation and the subsequent CFA revealed a four-factor model. However, four items showed cross-loadings or loaded on no factors. These items were removed from the model. The modified four-factor model consists of 20 items explaining 63.79% of the variance. In accordance with the Two-Dimensional Framework of Motivation (Forgeard and Mecklenburg, 2013), the first factor was named “Giving,” reflecting an extrinsic motivation that is oriented to others (e.g., helping others). The second factor, reflecting a self-orientated, intrinsic motivation (e.g., feeling curious), was named “Growth.” The third factor was named “Gain,” corresponding to an extrinsic, self-oriented, and even malevolent motivation (e.g., avoiding rules). The fourth factor is an additional intrinsic-self motivation called “Distraction” (e.g., killing time). The CFA provided satisfactory model fit for this factor solution (see Appendix). The internal consistency of the measures was good to excellent in both China (Cronbach’s α of 0.89 for Giving, 0.82 for Growth, 0.79 for Gain, and 0.73 for Distraction) and Germany (Cronbach’s α of 0.90 for Giving, 0.85 for Growth, 0.82 for Gain, and 0.79 for Distraction).

**Measures of Creativity Level** were developed based on a preliminary study involving Chinese and German participants that analyzed the content of 77 creative ideas that people posted in social media. We applied the eight domains of the Inventory of Creative Activities and Achievements (ICAA) (Diedrich et al., 2018) to code the posts, as it is among the very few creativity inventories that has also included the domain of Sports. Based on the preliminary data, we added three items related to Learning and Personal Development (knowledge acquisition, digital activities, personal growth), two items related to Social Engagement (virtual community, virtual parties), and two items related to Social Responsibility (charity, pro-social behavior). In addition, we also included two Malevolent Creativity items (mischief, personal gains) to reflect the fact that in times of crisis (such as the pandemic) some darker aspects of creativity might take place (Cropley et al., 2008; Cropley et al., 2014). Participants rated their level of creativity in each of the 14 domains on a 5-point Likert scale, with 1 representing “not at all creative” and 5 representing “extremely creative.”

Given that the focus of the current study is the change in the creativity level due to lockdown, participants were asked to rate their level of creativity twice: before and during the lockdown period. Following previous studies (e.g., Ding et al., 2015; Renner et al., 2017), we used the difference score of creativity before and during the lockdown as the measure of the change in creativity in subsequent analyses. An EFA (with Varimax rotation) using the delta scores of the 14 creative engagement items before and during the lockdown suggested a three-factor model accounting for 58.36% of the variance. Four items were excluded from this model due to non- or cross-loadings. However, the results of the CFA indicated a better fit for a two-factorial structure in both countries, which does not contain the two malevolent items. These items were further excluded from the model and the two-factor model, composed of creativity in Arts (e.g., singing, painting, cooking, handcrafting) and Learning and Ideas (e.g., attending webinars, creating business ideas) was maintained. The internal consistency of the measures was not very high yet acceptable in comparison to other measures of the study, with Cronbach’s α between 0.62 and 0.79 in China and between 0.62 and 0.67 in Germany.

### Measure Invariance Test

Although results indicated an acceptable fit of the factorial structure for the measurements in each country, we conducted measurement invariance tests to ensure that the scores and their interpretation were comparable in different cultural settings. Following Putnick and Bornstein (2016), we tested whether the loading patterns on the different latent factors were similar in each country (i.e., configural invariance), and whether the contribution of each item was similar to the factors for each country (i.e., metric invariance). To evaluate the model fit we used the cut-off criterion of a –0.01 change in CFI, and a RMSEA change of 0.015 (Cheung and Rensvold, 2002; Chen, 2007). As Table 3 shows, the configural invariance scores of the three variables reached a satisfactory level and the factorial structure of the constructs was consistent in both countries. However, due to partial metric non-invariance, the loadings of items are not fully equivalent across both countries. Thus, we tested partially invariant models and found that partial metric invariance existed for activating negative mood (ANM) and creativity level in Arts and Learning and Ideas.

**TABLE 3:** Measurement invariance test of the variables under investigation across the two countries.

Model	χ^2^ (df)	CFI	RMSEA	Δχ^2^ (Δdf)	ΔCFI	ΔRMSEA
**Activating Positive and Negative Mood**						
Configural Invariance	30.467 (16)	0.990	0.049	–	–	–
Metric Invariance	53.185 (20)	0.978	0.066	22.718 (4)	–0.012	0.017
Partial Metric Invariance	31.113 (19)	0.992	0.041	0.646 (3)	0.002	–0.008
**Motivations for Creativity during lockdown**						
Configural Invariance	975.653 (302)	0.917	0.049	–	–	–
Metric Invariance	1036.662 (318)	0.911	0.066	61.009 (16)	–0.006	0.017
**Creativity Level**						
Configural Invariance	74.057 (36)	0.967	0.049	–	–	–
Metric Invariance	102.851 (42)	0.948	0.066	28.794 (6)	–0.019	0.017
Partial Metric Invariance	88.700 (40)	0.958	0.041	14.643 (4)	–0.009	–0.008

Taken together, the results of cross-cultural equivalence tests indicate that a comparison of the observed constructs seems appropriate across countries; however, due to partial metric invariance for creativity level and ANM, any direct comparison (e.g., mean differences between Germany and China) has to be interpreted with caution (Putnick and Bornstein, 2016). As the focus of the current study is not the direct comparison of the two countries in individual variables, rather, the comparison of the associations among the variables, this partial metric invariance will not pose severe challenge for the further data analysis.

## Results

### Correlation Analysis

Both APM and ANM were positively correlated with all motivation variables to a moderate degree with *r* ranging from 0.14 to 0.45, *p* < 0.01. All correlations between moods and motivations were significant across Germany and China. In the Chinese sample, ANM was only associated with change in creativity in Learning and Ideas (*r* = 0.10, *p* < 0.05). In the German sample, APM was positively correlated with the change in creativity level in Arts (*r* = 0.15, *p* < 0.01) and Learning and Ideas (*r* = 0.20, *p* < 0.001). In both countries, the Giving, Growth and Distraction motivations were positively correlated to change in creativity Arts and Learning and Ideas. Differences were found in the Gain motivation in that this motivation was not correlated to the change in creativity in any creative activities in the Chinese sample, and negatively related to the change in creativity in Leaning and Ideas in the German sample. Table 4 presents the results of descriptive statistics and correlations of all variables.

**TABLE 4:** Descriptive statistics, correlations and reliability of variables.

Variables	1	2	3	4	5	6	7	8	9	10	11
**Chinese sample (*n* = 415)**											
(1) Age	(–)										
(2) PICL	–0.01	(–)									
(3) PICW	–0.01	0.67***	(–)								
(4) APM	–0.01	0.04	0.01	(0.82)							
(5) ANM	–0.05	0.40***	0.40**	0.10*	(0.83)						
(6) Giving	−0.11*	0.09	0.10*	0.43***	0.21***	(0.89)					
(7) Growth	−0.12*	0.04	0.04	0.41***	0.16**	0.74***	(0.82)				
(8) Gain	−0.12**	0.16**	0.13**	0.25***	0.29***	0.30***	0.27***	(0.79)			
(9) Distraction	−0.21***	0.21***	0.20***	0.21***	0.44***	0.45***	0.50***	0.27***	(0.73)		
(10) Artistic Creativity (Δ)	–0.07	0.12*	0.08	0.04	0.08	0.20***	0.22***	–0.00	0.19***	(–)	
(11) Learning and Ideas (Δ)	−0.10*	0.08	0.06	–0.03	0.10*	0.11*	0.19***	0.01	0.12*	0.48***	(–)
**German sample (*n* = 339)**											
(1) Age	(–)										
(2) PICL	–0.05	(–)									
(3) PICW	–0.05	0.64***	(–)								
(4) APM	−0.14**	0.13**	0.05	(0.73)							
(5) ANM	−0.14**	0.41**	0.27***	0.12*	(0.75)						
(6) Giving	–0.09	0.17**	0.14*	0.41***	0.29***	(0.90)					
(7) Growth	–0.06	0.19***	0.17**	0.45***	0.21***	0.78***	(0.85)				
(8) Gain	−0.11*	0.07	0.01	0.24***	0.24***	0.40***	0.27***	(0.82)			
(9) Distraction	−0.24***	0.29***	0.26***	0.14**	0.44***	0.39***	0.40***	0.18**	(0.79)		
(10) Artistic Creativity (Δ)	−0.13*	0.06	0.08	0.15**	–0.02	0.15**	0.19**	–0.07	0.13*	(–)	
(11) Learning and Ideas (Δ)	−0.19**	0.09	0.15**	0.20***	0.00	0.16***	0.21***	−0.14**	0.16**	0.48***	(–)

*p < 0.05, **p < 0.01, ***p < 0.001. *M*, mean; *SD*, standard deviation. The value in the () is Cronbach’s a; Artistic Creativity and Learning and Ideas are Δ scores of change in creativity before and during COVID-19 lockdown. PICL, Perceived Impact of COVID-19 on Private Life; PICW, Perceived Impact of COVID-19 on Working Life; APM, Activating Positive Mood; ANM, Activating Negative Mood. Gain was transformed using LG10(K-X) for the Chinese and German data. This transformation was conducted following the recommendation of Tabachnik and Fidell (2007, p. 89) to cope with the substantial negative skewness of the variable in the data.

### Comparison of Creative Level Before and During Lockdown

Paired sample *t*-tests were conducted in Chinese and German samples respectively to compare the creative level before and during lockdown. On applying the Bonferroni correction, the cutoff for the significance level was 0.0025 in both countries. As Table 5 shows, there was a significant increase in creativity in all everyday activities in both countries with only two exceptions in the German sample (in visual and performing arts). In the Chinese participants, the strongest increase was in knowledge acquisition, *t*(414) = 16.98, *d* = 0.83, followed by the increase in culinary arts, *t*(414) = 14.33, *d* = 0.70, *p* < 0.001 for all activities. Among the German participants, the biggest increase of creativity was found in social online engagement, *t*(338) = 11.30, *d* = 0.61 followed by knowledge acquisition, *t*(338) = 6.45, *d* = 0.35, *p* < 0.001 for both.

**TABLE 5:** Means (standard deviations) and paired sample *t*-test results for the comparison between the creativity level in different domains before and during lockdown in both countries.

Creativity Level	Mean (*SD*)		*t*	*p*	*d*
	
	Before COVID-19	During			
**China (*n* = 415)**					
*Arts (total score)*	*10.27 (3.59)*	*12.60 (4.05)*	*13.96*	*0.000*	*0.69*
Culinary Arts (e.g., cooking)	2.40 (1.03)	3.19 (1.11)	14.33	0.000	0.70
Visual Arts (e.g., painting)	1.95 (0.94)	2.32 (1.06)	9.08	0.000	0.45
Performing Arts (e.g., singing)	2.01 (0.96)	2.28 (1.11)	5.72	0.000	0.28
Crafts (e.g., handcrafting)	1.96 (0.93)	2.43 (1.17)	10.30	0.000	0.51
Digital Activities (e.g., making videos)	1.94 (1.02)	2.38 (1.22)	10.47	0.000	0.51
*Learning and Ideas (total score)*	*7.12 (2.31)*	*8.59 (2.59)*	*12.45*	*0.000*	*0.61*
Knowledge Acquisition (e.g., webinars)	2.51 (0.98)	3.40 (1.05)	16.98	0.000	0.83
Social Online-Engagement (e.g., calls)	2.38 (1.10)	2.65 (1.23)	3.85	0.000	0.19
Inventions (e.g., business ideas)	2.24 (0.99)	2.54 (1.13)	9.06	0.000	0.45
**Germany (*n* = 339)**					
*Arts (total score)*	*9.97 (3.49)*	*10.74 (3.74)*	*5.81*	*0.000*	*0.32*
Culinary Arts (e.g., cooking)	2.90 (1.06)	3.23 (1.12)	6.06	0.000	0.33
Visual Arts (e.g., painting)	1.75 (1.11)	1.79 (1.13)	1.31	0.190	0.07
Performing Arts (e.g., singing)	1.75 (1.11)	1.73 (1.11)	–0.43	0.670	0.02
Crafts (e.g., handcrafting)	1.88 (1.02)	2.14 (1.23)	5.86	0.000	0.32
Digital Activities (e.g., making videos)	1.69 (1.06)	1.85 (1.27)	4.18	0.000	0.23
*Learning and Ideas (total score)*	*5.74 (2.42)*	*6.90 (2.90)*	*10.52*	*0.000*	*0.57*
Knowledge Acquisition (e.g., webinars)	1.99 (1.05)	2.34 (1.29)	6.45	0.000	0.35
Social Online-Engagement (e.g., calls)	1.89 (1.04)	2.55 (1.32)	11.30	0.000	0.61
Inventions (e.g., business ideas)	1.86 (2.00)	2.00 (1.12)	3.73	0.000	0.20

### The Influence of Moods and Motivation on the Change in Creativity Level

To further investigate the influence of moods and motivation on the change in creativity, we conducted hierarchical regression analyses for each country with change in creativity in Arts and Learning and Ideas as dependent variables. In the first step, age and perceived influence of COVID-19 on private lives and work were entered into the model as control variables. In the second step, the two mood variables (APM and ANM) were added and then, in the last step, the motivational variables (Giving, Growth, Gain, and Distraction) were entered into the model. Table 6 shows that with Chinese participants, moods alone did not contribute to the change in creativity in the artistic domain. The inclusion of the motivational variables, however, increased the explained variance significantly, Δ*R*^2^ = 0.06, *p* < 0.001. Considering the change of creativity in Learning and Ideas, entering motivational variables also contributed an additional amount of variance, Δ*R*^2^ = 0.04, *p* < 0.001. After all mood and motivational variables were entered to the model, APM turned out to be negatively associated with Learning and Ideas (β = –0.11, *p* < 0.05), whereas the Growth motivation (β = 0.26, *p* < 0.001) was positively related to the change in creativity in Learning and Ideas.

**TABLE 6:** Results of hierarchical regression analysis predicting change in creativity in Arts and Learning and Ideas during COVID-19 lockdown in China.

	Arts		Learning and Ideas	
		
	β	*R* ^2^	β	*R* ^2^
**Step 1**				
Age	–0.06		−0.10*	
PICL	0.12		0.07	
PICW	–0.01		0.02	
Total *R*^2^		0.02		0.02
Adjusted *R*^2^		0.01		0.01
Δ*R*^2^		0.02		0.02
**Step 2**				
Age	–0.06		–0.09	
PICL	0.11		0.05	
PICW	–0.01		–0.00	
APM	0.03		–0.04	
ANM	0.04		0.08	
Total *R*^2^		0.02		0.02
Adjusted *R*^2^		0.01		0.01
Δ*R*^2^		0.00		0.00
**Step 3**				
Age	–0.03		–0.08	
PICL	0.12		0.06	
PICW	–0.02		0.00	
APM	–0.06		−0.11*	
ANM	–0.01		0.07	
Giving	0.11		–0.04	
Growth	0.14		0.26**	
Gain	–0.10		–0.06	
Distraction	0.08		–0.02	
Total *R*^2^		0.08***		0.06**
Adjusted *R*^2^		0.06***		0.04**
ΔR^2^		0.06***		0.04**

*p < 0.05, **p < 0.01, ***p < 0.001. PICL, Perceived Impact of COVID-19 on Private Life; PICW, Perceived Impact of COVID-19 on Working Life; APM, Activating Positive Mood; ANM, Activating Negative Mood.

With the German sample (see Table 7), APM was positively related to change in creativity in Learning and Ideas; however, for Arts, the effect of APM was significant only without motivations being added to the model (β = 0.14, *p* < 0.05). When regressing change in creativity in Arts on moods, motivational variables additionally explained a significant amount of variance (Δ*R*^2^ = 0.04, *p* < 0.01) with the significant influence of Gain motivation (β = –0.16, *p* < 0.01). When regressing creativity in Learning and Ideas on moods, the Δ*R*^2^ at step 3 was 0.07, *p* < 0.001. Besides APM (β = 0.16, *p* < 0.01), the Gain motivation showed a negative effect (β = –0.25, *p* < 0.001). In summary, the consistent finding across the two samples was that mood alone did not necessarily lead to a change in creativity, but the addition of motivational variables into the model contributed to a significant additional amount of the variance.

**TABLE 7:** Results of hierarchical regression analysis predicting change in creativity in Arts and Learning and Ideas during COVID-19 lockdown in Germany.

	Arts		Learning and Ideas	
		
	β	*R* ^2^	β	*R* ^2^
**Step 1**				
Age	−0.13*		−0.18**	
PICL	0.02		–0.01	
PICW	0.06		0.15*	
Total *R*^2^		0.02		0.05***
Adjusted *R*^2^		0.01		0.05***
Δ*R*^2^		0.02		0.05***
**Step 2**				
Age	−0.12*		−0.16**	
PICL	0.03		–0.01	
PICW	0.07		0.16*	
APM	0.14*		0.18**	
ANM	–0.08		–0.08	
Total *R*^2^		0.04**		0.09***
Adjusted *R*^2^		0.03**		0.08***
Δ*R*^2^		0.02*		0.04**
**Step 3**				
Age	−0.12*		−0.17**	
PICL	0.02		–0.02	
PICW	0.03		0.12	
APM	0.09		0.16**	
ANM	–0.10		–0.08	
Giving	0.08		0.09	
Growth	0.10		0.10	
Gain	−0.16**		−0.25***	
Distraction	0.07		0.08	
Total *R*^2^		0.09***		0.16***
Adjusted *R*^2^		0.06***		0.14***
Δ*R*^2^		0.04**		0.07***

*p < 0.05, **p < 0.01, ***p < 0.001. PICL, Perceived Impact of COVID-19 on Private Life; PICW, Perceived Impact of COVID-19 on Working Life; APM, Activating Positive Mood; ANM, Activating Negative Mood.

### Mediation Analysis of the Mood-Creativity Relationship Through Motivation

To further examine the role of motivation in the Mood-Creativity link, we conducted mediation analyses using moods as independent variable, the change in creativity as dependent variable, and motivational variables as mediators, following the implications of some recent theories (e.g., De Dreu et al., 2012; Gendolla, 2017). Age and the perceived impact of COVID-19 on private and professional lives were entered as covariates. As Table 8 shows, with the Chinese sample, the only significant direct effect of moods was found between the APM and the change in creativity in the Learning and Idea domain controlling for the effect of the Growth motivation. The inclusion of the motivational variables in the model as mediators enabled more significant Mood-Creativity relationships in the Arts domain than the Learning and Ideas domain. In the Arts domain, all motivational variables mediated the Mood-Creativity relationships (except Gain motivation), regardless of whether it was under the APM or ANM conditions. In the Learning and Ideas domain, the Growth motivation mediated the Mood-Creativity relationship in both APM and ANM circumstances. The Giving motivation intervened the Mood-Creativity relationship in APM circumstances.

**TABLE 8:** Results of the mediation analysis of the effects of moods on change in creativity through motivation in China (*n* = 415).

						Indirect effect		
	
		a-Path	b-Path	c-Path	c’-Path	IE	CI (95%)	
							
IV	*M*	β	β	β	β	β	Lower	Upper
**Creativity in Arts (DV)**								
APM	Giving	0.43***	0.21**	0.04	−0.06	**0.09**	0.03	0.16
APM	Growth	0.41***	0.23***	0.04	−0.06	**0.10**	0.05	0.15
APM	Gain	0.24***	−0.04	0.04	0.05	−0.01	−0.04	0.02
APM	Distraction	0.20***	0.16**	0.04	0.00	**0.03**	0.01	0.06
ANM	Giving	0.20**	0.19**	0.04	0.00	**0.04**	0.01	0.08
ANM	Growth	0.17**	0.21**	0.04	0.00	**0.04**	0.01	0.06
ANM	Gain	0.26***	−0.04	0.04	0.05	−0.01	−0.04	0.02
ANM	Distraction	0.41***	0.18**	0.04	−0.03	**0.07**	0.03	0.12
**Learning and Ideas (DV)**								
APM	Giving	0.43***	0.13*	−0.03	−0.08	**0.06**	0.01	0.11
APM	Growth	0.41***	0.23***	−0.03	−0.12*	**0.09**	0.05	0.14
APM	Gain	0.24***	−0.01	−0.03	−0.02	−0.00	−0.03	0.02
APM	Distraction	0.20***	0.10	−0.03	−0.05	0.02	−0.00	0.04
ANM	Giving	0.20**	0.09	0.07	0.05	0.02	−0.00	0.05
ANM	Growth	0.17**	0.17**	0.07	0.04	**0.03**	0.01	0.05
ANM	Gain	0.27***	−0.04	0.07	0.08	−0.01	−0.04	0.02
ANM	Distraction	0.41***	0.07	0.07	0.04	0.03	−0.02	0.08

IV, independent variable; M, mediator variable; IE, indirect effect; CI, confidence interval; 5.000 bootstrap samples; significant indirect effects in bold. *p < 0.05, **p < 0.01, ***p < 0.001.

With the German sample (see Table 9), it was found that in circumstances of the APM, only a significant indirect effect was found for the Growth motivation mediating the Mood-Creativity relationship in the artistic domain. When Growth was entered as a mediator, APM was not associated with the change in creativity anymore. In circumstances of the ANM, all motivational variables except the Gain motivation intervened the Mood-Creativity relationship in the Arts domain. In the Learning and Ideas domain, under the APM conditions, only the Gain motivation mediated the Mood-Creativity relationship. Under the conditions of the ANM, all motivational variables mediated the Mood-Creativity relationship.

**TABLE 9:** Results of the mediation analysis of the effects of moods on change in creativity through motivation in Germany (*n* = 339).

						Indirect effect		
	
		a-Path	b-Path	c-Path	c’-Path	IE	CI (95%)	
							
IV	*M*	β	β	β	β	β	Lower	Upper
**Creativity in Arts (DV)**								
APM	Giving	0.39***	0.09	0.13*	0.09	0.04	−0.01	0.09
APM	Growth	0.44***	0.14*	0.13*	0.07	**0.06**	0.01	0.12
APM	Gain	0.22**	−0.12*	0.13*	0.16*	−0.03	−0.07	0.001
APM	Distraction	0.08	0.08	0.13*	0.12*	0.01	−0.00	0.02
ANM	Giving	0.26***	0.16**	−0.07	−0.11	**0.04**	0.01	0.08
ANM	Growth	0.15**	0.19**	−0.07	−0.10	**0.03**	0.01	0.06
ANM	Gain	0.25**	−0.08	−0.07	−0.05	−0.02	−0.06	0.01
ANM	Distraction	0.36***	0.13*	−0.07	−0.12	**0.05**	0.01	0.09
**Learning and Ideas (DV)**								
APM	Giving	0.39***	0.07	0.18**	0.15*	0.03	−0.02	0.08
APM	Growth	0.44***	0.13	0.18**	0.12	0.06	−0.01	0.12
APM	Gain	0.22**	−0.22**	0.18**	0.23**	**−0.05**	−0.09	−0.01
APM	Distraction	0.08	0.07	0.18**	0.17**	0.01	−0.00	0.02
ANM	Giving	0.26***	0.15**	−0.07	−0.11	**0.04**	0.01	0.08
ANM	Growth	0.15**	0.19**	−0.07	−0.10	**0.03**	0.01	0.07
ANM	Gain	0.25**	−0.16**	−0.07	−0.03	**−0.04**	−0.08	−0.01
ANM	Distraction	0.36***	0.13*	−0.07	−0.12	**0.05**	0.01	0.10

IV, independent variable; M, mediator variable; IE, indirect effect; CI, confidence interval; 5.000 bootstrap samples; significant indirect effects in bold. *p < 0.05, **p < 0.01, ***p < 0.001.

Taken together, it was found that the direct effect of APM on creativity is much more limited with the Chinese participants in comparison to the German participants. In the German sample, APM can directly lead to the change in creativity in both Arts and Learning and Ideas domains, but such direct Mood-Creativity paths cannot be observed for the ANM. Instead, motivations come into play as mediators to enable the Mood-Creativity relationship in both Arts and Learning and Ideas domains. Consistent with the findings from the German participants, no direct effect of the ANM on creativity was found among the Chinese participants; only when motivation entered the models as mediators more significant relations were found.

## Discussion

### Are People More or Less Creative in the Period of COVID-19 Lockdown?

The first research question of this study was whether people were more or less creative in the period of COVID-19 lockdown. It was found that in both countries, participants reported significant increases in creativity across all everyday activities, with the sole exception of visual and performing arts, where the change among the German participants was not significant. Chinese participants reported significant increases in creativity in all everyday activities with the strongest increase in knowledge acquisition followed by culinary art. These results may be attributed to the Chinese value of attaching great importance to seeking knowledge, cultivating a passion for lifelong learning, fostering diligence, and feeling “shame-guilt” for lack of desire to learn (Li, 2002). In times of lockdown, Chinese workers embraced this value and applied their creativity towards acquiring new knowledge. The increase in creativity in culinary arts among the Chinese participants is not surprising as food is an essential part of the Chinese society — as one Chinese saying goes, “Food is the paramount necessity of the people” (民以食为天). According to the Intelligence Current Theory (Shi, 2004), this positive attitude toward food could help the Chinese workers connect their “active intelligence” to food-related activities including cooking, thus increasing the change of the occurrence of creativity. Among the German participants, the biggest increase of creativity was found in social online engagement. With the lockdown, Germany experienced a rapid increase in the usage of digital technologies and services in areas such as digital health (Gerke et al., 2020) or online learning at universities (Skulmowski and Rey, 2020). The basis of this digitalization processes is in many areas the use of video calls over the internet. The Federal Statistical Office in Germany (2020)^2^ reported a 9% increase in the 1st quarter of 2020 in the usage of video and phone calls via internet compared to 2019. Hence, the usage of online video calls played an important role in various everyday situations during the lockdown in Germany.

### Cultural Differences in Mood and Creativity

Activating positive mood (APM) was found to be one of the most pronounced and consistent predictors of change in creativity during COVID-19 times in Germany, especially for Learning and Ideas. Although this finding is consistent with past work (To et al., 2012), Chinese participants showed almost no direct influence through APM on their change in creativity. Contextual and cultural factors play an important role in the emotion processes (Greenaway et al., 2018). This result might be explainable through varying cultural traditions forming different perceptions and value judgments of emotions (Averill and Sundararajan, 2006). De Vaus et al. (2018) argued that the acceptance of contradictions, a key component in holistic thinking, leads people to not value positive emotions over negative ones, and hence reduce the tendency to regulate emotions (e.g., reduce negative emotions). Furthermore, research indicates that low arousal emotions are preferred and experienced more in the East compared to the West (Lim, 2016). Our mood measures were based on activating moods which had a higher arousal. Hence, they might be less pronounced in the Chinese sample.

Among the German sample, APM, but not ANM, was positively related to the change of creativity in Learning and Ideas and partially in Arts. This finding aligns with a meta-analysis study which found that creativity is enhanced mostly by activated positive emotional states that are associated with an approach motivation and promotion focus (e.g., enthusiasm) (Baas et al., 2008). According to the dual pathway model of creativity, APMs lead to higher cognitive flexibility and inclusiveness, thus promoting creative performance (De Dreu et al., 2008). However, the hypothesis that ANMs could promote creativity was not supported in the present study. One reason might be the reinforcement of a flexibility strategy by Western institutions (Morris and Leung, 2010). Moreover, results of experiencing-sampling studies came to a similar conclusion, that active positive emotions significantly predicted day-to-day variability of creative activities (Silvia et al., 2014; Karwowski et al., 2017). As shown in Conner and Silvia’s (2015) daily diary study, high-activation positive emotions are the most favorable toward everyday creativity, whereas negative emotions are unrelated, or even antagonistic, with creativity. In turn, everyday creativity leads to increased well-being and flourishing on the next day (Conner et al., 2016), suggesting a possible upward spiral for creativity and positive affect. These findings reflect our results showing that the correlation between APM and everyday creativity is more significant than that between ANM and creativity.

Negative moods cannot explain the growth of creativity in Arts and Learning & Ideas even though the dual pathway model of mood suggests ANMs can improve cognitive persistence over time (To et al., 2012). Given that we tested participants’ moods and creative activities concurrently, this type of long-term effect was not present. On the other hand, such negative affect would lead to bottom–up information processing, in which people focus on details of the external environment to overcome such negative situations, as opposed to participating in recreational activities (Clore et al., 2001). Therefore, we cannot explain the growth of creativity in Arts and Learning and Ideas through ANMs.

In terms of Chinese participants, neither APM nor ANM alone explained the increase of creativity in everyday activities. Chinese culture tends to hold a holistic view of emotions, making it difficult to separate them into positive and negative ones (De Vaus et al., 2018). Moreover, Chinese people tend to develop refined emotions that cannot be expressed specifically (Averill et al., 2001; Averill and Sundararajan, 2006). Among others, Frijda and Sundararajan (2007) suggested that when the Chinese experience bad feelings, they tend to analyze and find meanings from these experiences through self-reflection and develop their life attitudes. As a result, it is the meanings that are obtained from the analyses of emotions that lead to the engagement of creative activities. Thus, two kinds of activating moods cannot provide an explanation for their increase in creativity in everyday activities. It is possible that future studies focused on meaning-making and creativity may yield additional information (Kaufman, 2018).

We can thus suggest that during a negative experience, the Mood-Creativity relationship reflected cultural differences. People in Eastern cultures tend to emphasize authentic or useful evaluations of creativity and express their emotions through an intrapersonal way. In contrast, those in Western cultures are more likely to have an interpersonal orientation and evaluate creativity focusing on novelty, thereby valuing the people who are good at expressing their emotions (Averill et al., 2001; Averill and Sundararajan, 2006; Morris and Leung, 2010). Masuda et al. (2008) also found that people who live in a collectivist culture perceive emotions through social contexts and others’ expressions of emotion, whereas Western people see emotions as individuals’ expressions. In terms of mood regulation, when people are primed to feel mortality salience, Eastern cultures tend to hold a holistic view on positive and negative moods and think dialectically, which leads to a flexible way to regulate and cope with emotions (De Vaus et al., 2018). Furthermore, Eastern Asians are inclined to engage in and enjoy their daily life (Ma-Kellams and Blascovich, 2012), thereby lifting themselves out of bad moods. Taken together, the expression of mood is refined in Chinese samples and they tend to regulate negative moods flexibly and seek enjoyable daily life even during a crisis. In contrast, German participants tend to express their moods in a more private and intense fashion such that moods more easily influence creative engagement.

### The Mediating Role of Motivation in the Mood-Creativity Relationship

The results of the hierarchical regression analysis show that adding motivation to the Mood-Creativity equation helps explain an additional amount of variance in both countries. Furthermore, results of the mediation analysis indicate that positive or benevolent motivations (e.g., Growth, Giving, or Distraction) help explain the Mood-Creativity relationship, especially in circumstances of negative mood states. This relationship was more pronounced in creative activities involving learning or idea generation. These findings are to some extent contrary to the existing body of literature emphasizing the positive mood-creativity relationship (e.g., Baas et al., 2008; Davis, 2009); however, they are in line with past findings that suggest the beneficial influence of positive mood is not equal for every person and situation (Akbari Chermahini and Hommel, 2012). Moreover, our findings empirically validate the recommendations by researchers (e.g., Kaufmann, 2003a) to consider a wider variety of positive moods when examining complex situations. Our results show that the Mood-Creativity relationship is strongly dependent on specific motivations that are triggered through the different mood states and hence explain a negative-mood-creativity relationship. These findings echo the call of Martin et al. (1993) to consider different motivations that can be triggered through moods. Our results show that the effects of negative mood on creativity through motivation are much more pronounced in creative activities that involve learning and idea generation during COVID-19. This might be explained by the novel situation provoked by COVID-19, which often required finding creative solutions to problems caused by the lockdown or restrictions. Negative moods increase people’s motivation to act, particularly when adaptation is required (Forgas, 2013). Furthermore, in situations where creative problem solving is required, negative mood states can be beneficial for creativity (Kaufmann, 2003b). Thus, our results add to the existing body of literature by emphasizing the consideration of motivation in both positive and negative Mood-Creativity relationships, especially in new situations where creativity with a learning or problem-solving orientation is required. In the current study, positive moods were positively related to people’s motivation to be creative in order to help others (i.e., Giving) and improve themselves (i.e., Growth) in the Arts domain. Negative moods were associated to a higher Distraction motivation for creativity. This coping function of creativity has shown to help relieve anxiety (Grossman, 1981), trauma (Forgeard, 2013), and emotion regulation (Fancourt et al., 2019). Furthermore, Mann and Cadman (2014) showed that boredom, which many people faced during the COVID-19 lockdown, can help people foster their creativity.

### Limitations and Future Studies

The present study is not without limitations. First, though the retrospective approach is an established method in psychological studies and has been widely used in studies related to crisis and negative events (e.g., Jacobs, 2002; Couch and Olson, 2016; Lord et al., 2019) including some related to the consequences of the COVID 19 pandemic (e.g., Jungmann and Witthft, 2020; Manuela and Gabriella, 2021), this method poses limitations to the accurate assessment of moods and motivation. Given that mixed feelings can decline or decay in memory, future studies should consider using in-the-moment emotion assessment (Moeller et al., 2018). Such methods may include a diary study or the experience sampling method (ESM, Silvia et al., 2014) to further explore the fluctuation of emotion and motivation in times of crisis. Secondly, the creative activities examined in this study were developed based on a small-scale preliminary study using an inventory (i.e., ICAA, Diedrich et al., 2018) developed with Western samples. One risk is that it is possible that some creative activities that non-Western cultures may have engaged in were underrepresented. Future studies should consider adding open-ended questions to supplement existing measurements of creative engagement. Last but not least, this study mainly focused on the emotions and motivations underlying the creative behaviors in this pandemic; however, the antecedent variables of emotions and motivations were not addressed. It is worth examining whether some specific events during the quarantine may have triggered particular emotions or motivations. Lades et al. (2020) found that certain daily life events (e.g., exercising, taking care of children) were associated with raised positive affect and reduced negative affect. In future studies, context-related variables can be included as possible antecedent variables of emotions, motivations, and creative behaviors, so as to get a deeper understanding of the pandemic’s impact on people’s creative engagement.

## Conclusion

COVID-19 and its subsequent consequences (e.g., restrictions or lockdowns) have had a huge impact on our daily habits, relationships, and professional careers. In such a new situation, we have to change, adapt, and rethink the way of life we led before the pandemic. Creativity plays an important role in helping us doing so. Despite cultural differences and different subjective feelings toward the pandemic, results of this study show that we have one thing in common: when crises like the COVID-19 pandemic occur, we often turn to creativity to help us grow, give back, and get distracted. This study contributes to the existing body of research by showing the importance of not only considering positive or negative moods when talking about creative behaviors, but also investigating how motivational states intervene in mood states to enable creativity.

## Data Availability Statement

The raw data supporting the conclusions of this article will be made available by the authors upon request.

## Ethics Statement

The studies involving human participants were reviewed and approved by The Institutional Review Board of the University of Applied Management, Germany and the Institutional Review Board of the Nankai University, China. The patients/participants provided their written informed consent to participate in this study.

## Author Contributions

All the authors participated in the research design and the adaption of the measures. SH analyzed the data and participated in the writing of the manuscript. XZ collected the data in China and participated in the writing of the manuscript. MT coordinated the international cooperation and participated in the writing of the manuscript. CW collected the data in Germany. JK edited the final draft of the manuscript.

## Conflict of Interest

The authors declare that the research was conducted in the absence of any commercial or financial relationships that could be construed as a potential conflict of interest.

## Publisher’s Note

All claims expressed in this article are solely those of the authors and do not necessarily represent those of their affiliated organizations, or those of the publisher, the editors and the reviewers. Any product that may be evaluated in this article, or claim that may be made by its manufacturer, is not guaranteed or endorsed by the publisher.

## Footnotes

1 The logic commonly provided was that the COVID-19 pandemic is a natural disaster. No single persons are responsible for this pandemic therefore, there is no reason to feel excited or ashamed of this pandemic.

2
https://www.destatis.de/EN/Press/2020/08/PE20_302_639.html;jsessionid=9702E1C1DB7B7B8A398291B17B033A2E.internet8712

## Appendix

### Results of EFA and CFA of the Measures

#### (1) EFA and CFA for Positive and Negative Activating Moods

**TABLE 1.1:** EFA results for the pooled sample of the activating moods during lockdown based on a split sample.

	Factor 1:	Factor 2:
		
Items	Activating positive mood	Activating negative mood
Upset	**0.85**	0.11
Angry	**0.85**	0.06
Anxious	**0.80**	−0.01
Enthusiastic	0.12	**0.88**
Inspired	0.04	**0.86**
Interested	0.01	**0.74**

**n* = 286. Extraction method: Principal Component Analysis; Rotation method: Varimax with Kaiser normalization. Loadings > 0.40 are in bold.*

**TABLE 1.2:** CFA results for the pooled sample of the activating moods during lockdown based on a split sample.

Model	χ^2^ (df)	RMSEA	CFI	SRMR	TLI
**Activating positive and negative mood (two-factors)**					
China	21.07 (8)	0.08	0.98	0.04	0.96
Germany	24.63 (8)	0.10	0.95	0.05	0.91

**n* = 468.*

#### (2) EFA and CFA for Motivations for Creative Engagement

**TABLE 2.1:** EFA results for the pooled sample of motivations for creative engagement during lockdown based on a split sample.

	Factor 1:	Factor 2:	Factor 3:	Factor 4:
				
Items	Giving	Growth	Gain	Distraction
Cheering other people up	**0.92**	−0.05	−0.03	−0.06
Connecting with other people	**0.87**	−0.14	−0.02	0.11
Helping other people	**0.79**	0.14	−0.03	−0.14
Entertaining others	**0.76**	−0.10	0.11	0.06
Impressing other people	**0.67**	−0.06	0.24	0.01
Coping with stress	**0.60**	−0.07	−0.03	0.34
Sense of purpose	**0.55**	0.35	0.00	−0.21
Improving mood	**0.46**	0.37	−0.06	0.19
Feeling curious	−0.31	**0.88**	0.04	0.17
Self-improvement	0.24	**0.69**	−0.06	−0.13
Self-expression	−0.04	**0.64**	0.22	0.05
Making sense of life	0.21	**0.57**	−0.08	0.09
Feeling good about myself	0.31	**0.56**	−0.08	−0.07
Sabotaging others	0.02	−0.17	**0.88**	0.07
Getting revenge on people	0.05	−0.04	**0.85**	−0.01
Avoiding rules	−0.03	0.13	**0.78**	−0.04
Becoming well-known	0.07	0.25	**0.65**	−0.05
Killing time	−0.01	−0.02	−0.03	**0.88**
Feeling bored	−0.07	0.05	0.07	**0.81**
Needing distraction	0.14	0.21	−0.06	**0.62**

**n* = 286. Extraction method: Principal Component Analysis; Rotation method: Oblimin with Kaiser normalization. Loadings > 0.40 are in bold.*

**TABLE 2.2:** CFA results for the pooled sample of motivations for creative engagement during lockdown based on a split sample.

Model	χ^2^ (df)	RMSEA	CFI	SRMR	TLI
Motivations for creative Engagement (four-factors)					
China	371.60 (151)	0.08	0.91	0.07	0.89
Germany	381.80 (151)	0.08	0.91	0.08	0.89

**n* = 468.*

#### (3) EFA and CFA for Change in Creativity Before and During Lockdown

**TABLE 3.1:** EFA results of the pooled sample of change in creativity before and during lockdown based on a split sample.

	Factor 1:	Factor 2:	Factor 3
			
Items	Artistic Creativity	Learning and Ideas	Malevolent Creativity^∗^
Visual arts (e.g., painting)	**0.79**	0.06	0.00
Performing arts (e.g., singing)	**0.78**	−0.04	−0.05
Culinary arts (e.g., cooking)	**0.74**	0.17	0.06
Crafts (e.g., handcrafting)	**0.73**	0.28	0.03
Digital Activities (e.g., making videos)	**0.58**	0.27	0.01
Social online-engagement (e.g., group calls)	−0.03	**0.74**	−0.04
Knowledge acquisition (e.g., webinars)	0.22	**0.67**	0.13
Inventions (e.g., business ideas)	0.36	**0.63**	0.02
Mischief (e.g., playing tricks)*	−0.05	−0.04	**0.84**
Personal gains (e.g., finding loopholes)*	0.06	0.12	**0.82**

**n* = 286. Extraction method: Principal Component Analysis; Rotation method: Varimax with Kaiser normalization. Loadings > 0.40 are in bold. *Not included in the final factorial structure.*

**TABLE 3.2:** CFA results of the pooled sample of change in creativity before and during lockdown based on a split sample.

Model	χ^2^ (df)	RMSEA	CFI	SRMR	TLI
**Change in creativity (two-factors)**					
China	56.89 (18)	0.09	0.92	0.06	0.87
Germany	25.33 (18)	0.04	0.97	0.04	0.96

**n* = 468.*
